# *Eurycoma longifolia* upregulates osteoprotegerin gene expression in androgen- deficient osteoporosis rat model

**DOI:** 10.1186/1472-6882-12-152

**Published:** 2012-09-12

**Authors:** Ahmad Nazrun Shuid, Eman El-arabi, Nadia Mohd Effendy, Halimaton Saadiah Abdul Razak, Norliza Muhammad, Norazlina Mohamed, Ima Nirwana Soelaiman

**Affiliations:** 1 Department of Pharmacology, Faculty of Medicine, National University of Malaysia (Universiti Kebangsaan Malaysia), Jalan Raja Muda Abd Aziz, 50300 , KL, Malaysia; 2 Department of Biomedical Science, Faculty of Health Sciences, National University of Malaysia (Universiti Kebangsaan Malaysia), Jalan Raja Muda Abd Aziz, 50300 , KL, Malaysia

**Keywords:** *Eurycoma longifolia*, Osteoporosis, Orchiectomy, OPG, RANKL

## Abstract

**Background:**

*Eurycoma longifolia* (EL) has been shown recently to protect against bone calcium loss in orchidectomised rats, the model for androgen-deficient osteoporosis. The mechanism behind this is unclear but it may be related to its ability to elevate testosterone levels or it may directly affect bone remodeling. The aim of this study is to determine the mechanism involved by investigating the effects of EL extract on serum testosterone levels, bone biomarkers, biomechanical strength and gene expression of Receptor Activator of Nuclear Factor kappa-B ligand (RANKL), Osteoprotegerin (OPG) and Macrophage-Colony Stimulating Factor (MCSF) in orchidectomised rats.

**Methods:**

Thirty-two male Sprague–Dawley rats were divided into: Sham-operated group (SHAM); orchidectomised-control group (ORX); orchidectomised and given 15 mg/kg EL extract (ORX + EL) and orchidectomised and given 8 mg/kg testosterone (ORX + T). The rats were treated for 6 weeks. The serum levels of testosterone, osteocalcin and C-terminal telopeptide of type I collagen (CTX) were measured using the ELISA technique. The femoral bones were subjected to biomechanical testing. The tibial bone gene expressions of RANKL, OPG and MCSF were measured using the branch DNA technique.

**Results:**

The post-treatment level of testosterone was found to be significantly reduced by orchiectomy (p < 0.05). Both ORX + EL and ORX + T groups have significantly higher post-treatment testosterone levels compared to their pre-treatment levels (p < 0.05). The bone resorption marker (CTx) was elevated after orchiectomy but was suppressed after treatment in the ORX + EL and ORX + T groups (p < 0.05). There was no significant finding for the femoral biomechanical parameters. The tibial OPG gene expression in the ORX group was significantly lower compared to the SHAM and ORX + EL groups (p < 0.05).

**Conclusion:**

Supplementation with EL extract elevated the testosterone levels, reduced the bone resorption marker and upregulated OPG gene expression of the orchidectomised rats. These actions may be responsible for the protective effects of EL extract against bone resorption due to androgen deficiency.

## Background

Osteoporosis in men is gaining more interests as it is becoming one of the main cause of morbidity and mortality in older men. Approximately 2 million men in the United States suffered from osteoporosis [1] and 1.5 million of them are more than 65 years old [2]. Osteoporosis is usually asymptomatic and commonly presents with fractures due to minimal trauma [3]. The mortality associated with hip fractures is higher in men than in women and men are twice likely to die following a hip fracture (14% versus 6% for women) [4]. It is estimated that 31% of men with hip fracture died within 1 year after fracture as compared to only 17% of women [5]. There are very few studies carried out on osteoporosis in males but the necessity for potential therapeutic options is increasing due to an increase in male life expectancy and the associated hypogonadism.

The main cause of osteoporosis in men is androgen deficiency due to natural aging [6]. Partial androgen deficiency is common in aged men as 20–30% of them suffered from testosterone deficiency, exposing them to a high rate of bone loss which may lead to osteoporosis [7]. The testosterone- deficient state was shown to promote bone resorption only, as the CTx levels were found to be elevated in orchidectomised rats but the osteocalcin remained unchanged. Furthermore, testosterone replacement prevented the CTx elevation induced by orchiectomy [8]. Human studies have shown that testosterone replacement therapy caused a reductions in bone resorptive markers [9-11] and increased calcium absorption and bone formation [12].

The most widely practiced means of testosterone therapy is by intramuscular injection. It provides immediate testosterone surge but is painful and associated with prostate cancer and liver dysfunction. The oral form of testosterone is largely converted into inactive metabolites by the liver and is associated with liver tumors. Transdermal testosterone in the form of cream and gel may be sticky and has a bad odour. There are also concerns regarding transference of the applied testosterone cream or gel to women and children by skin contact. This is also the most expensive form of testosterone as the concentration required may be up to 4 to 5 times higher than other preparations [13]. With all these problems with testosterone therapy, alternative treatment modalities that have testosterone-like action are required for treatment of testosterone deficiency and preventing the associated bone loss.

*Eurycoma longifolia (EL)*, classified under the *Simaroubaceae* family, is a tall and slow growing tree found in South-east Asian countries. The plant extract is widely used to enhance male sexuality in Asia. The active ingredients are called quassinoids and are found in the root [14]. Studies have confirmed its aphrodisiac and ergogenic effects [15-19]. Our previous study found that supplementation of *EL* to orchidectomised rats was able to maintain the bone calcium content. EL may have achieved this by modulating the bone resorptive activity of osteoclasts [8].

Many factors that affect bone resorption were found to do so by altering Receptor Activator of Nuclear Factor kappa-B ligand (RANKL) and Osteoprotegerin (OPG) production [20]. RANKL is a very important cytokine for differentiation and activation of osteoclasts [21]. It was reported that the administration of serum RANKL to mice promoted osteoclast growth and activation, leading to osteoporosis [22]. Furthermore, a human study by Stern et al., [23], found that high RANKL levels were associated with low bone mineral density (BMD) in men but no RANKL-BMD associations were found in women. They proposed that the inverse RANKL-BMD association in men may be related to testosterone levels.

The antiresorptive decoy receptor (OPG) opposes RANKL by binding with RANKL and preventing RANKL from binding to RANK receptors. As a result, OPG inhibits the osteoclastogenetic process and bone resorption. Osteoclastogenesis also requires Macrophage-Colony Stimulating Factor (MCSF), which is also expressed by osteoblasts. MCSF, binds to the MCSF receptors situated in the osteoclasts, but the mechanism to modulate osteoclastogenesis is still not clear [24].

To the best of our knowledge, there is no study on the mechanism of EL extract in preventing bone loss due to androgen deficiency. Originally, EL extract was used by men for its aphrodisiac effects, believed to be contributed by its ability to raise testosterone levels. A study has shown that EL extract was capable of enhancing testosterone production [25]. In order to confirm that the testosterone-raising ability of EL extract may be responsible for its effects on bone, the serum testosterone levels were measured in our rat model. The bone resorptive marker, CTX and bone formation marker, osteocalcin were measured to determine its effect on bone remodeling. The biomechanical strength of the bones was also assessed. As the differentiation and activation of osteoclast are influenced by the RANKL/OPG system and MCSF, their gene expressions were measured to determine the molecular mechanism of EL extract in protecting bone against androgen-deficient osteoporosis.

## Methods

### Animal model

Thirty two male Sprague Dawley rats, aged 10 months old and weighing between 250 to 300 grams were used. The study had been approved by the UKM Animal Ethics Committee (FP/FAR/2008/NAZRUN/13-FEB/217-FEB-2008-FEB-2010). The rats were divided into the following groups: sham-operated (SHAM), orchidectomised-control (ORX), orchidectomised and given testosterone replacement (ORX + T) and orchidectomised and supplemented with EL (ORX + EL). Middle-aged orchidectomised rats were used as the model for androgen-deficient osteoporosis [26]. Before performing orchiectomy, the rats were anesthetized with Ketapex:Xylazil (1:1). A 2-cm ventral midline incision was made in the scrotum and the tunica was pierced. The testes were pushed out and raised to expose the underlying blood vessels and tubules. The spermatic chord was clamped and tied with absorbable catgut suture at the confluence of the blood vessels and epididymis. The testes were removed and all deferential vessels and ducts were replaced back into the tunica. The tunica of the contralateral side was similarly penetrated and the procedure repeated. The scrotal incision was closed back with a suture.

#### *Eurycoma longifolia* extract

*Eurycoma longifolia* Jack extract was obtained from Phytes Biotek Sdn. Bhd. (Selangor, Malaysia), a licensed GMP manufacturer of herbal products, in the form of a freeze-dried standardized extract (Batch No: TA 071210). It was extracted using a patented high pressure water extraction process (US 7,132,117 B2), filtered at 1–4 micron and freeze dried without maltodextrin or lactose. Physically, it was a light brown fine powder with 4-6% moisture content. Its major chemical components were proteins (31.75%), glycosaponins (41.08%) and eurycomanone (1.604%). This extract was the same form used for human consumption as health supplements [27].

The EL aqueous extract powder was dissolved in normal saline and given via oral gavage at the doses of 15 mg/kg rat weight daily at 9 am for 6 weeks [25]. Testosterone was purchased from TCI UK Ltd (UK). It was diluted in olive oil (Bertolli, Italy) and 8 mg/kg was injected intramuscularly once daily at 9 am for 6 weeks [28].

Body weights were measured weekly. Blood samples were collected twice; before the start of treatment and after 6 weeks of treatment. They were obtained under anesthesia from the retro-orbital vein. The blood was then centrifuged at 2000 xg for 10 minutes and the serum stored at −70°C. At the end of treatment, the rats were euthanized and both tibiae and femora were removed, cleansed of all soft tissues and stored at −70°C.

### Bone biochemical markers

Bone biochemical markers of serum osteocalcin and C- terminal telopeptide of type I collagen (CTX) were measured using an ELISA reader (VERSAmax, Sunnyvale, USA). The kits used were the Rat Osteocalcin ELISA (Biomedical Technologies, Herlev, Denmark) and RatlapsTM ELISA CTX-1 kits (Nordic Biosciences, IDS UK). The testosterone levels were also measured using the Testosterone ELISA Kit ( i-DNA Biotechnology, Hamburg, Germany).

### Biomechanical testing

The femora were prepared for biomechanical testing. They were kept moist at all time by wrapping them with gauze soaked in phosphate buffered solution and aluminum foil. The study groups were numbered to blind the operators. Each femur was placed on the Instron machine (Instron Microtester 5848, Instron Corp., USA) in a three-point bending configuration. The load was applied at the mid-diaphysis in an anteroposterior direction with a loading speed of 5 mm/min until the femur fractured. The load, stress and strain-deflection curves were automatically calculated by the computer using the Bluehill software.

### Gene expression

The tibial bones were grounded into a fine powder with mortar and pestle with addition of liquid nitrogen. Proteinase K was added to release the ribonucleic acid (RNA) and prepared according to directions suggested by Panomics (Fremont, CA) for analysis of mRNA expression using the Panomics QuantiGene Plex 2.0 system. The method combines RNA signal amplification and microspheres with unique fluorescent signatures to enable quantitation of multiple mRNA targets simultaneously in the same sample, without having the amplification inaccuracies of RT-PCR, and allows for discrimination of highly homologous messages [29,30]. Specific oligonucleotide capture and extender probe sets (3 per target), designed to anneal exclusively to each mRNA of interest and each housekeeping mRNA, were designed by Panomics to unique sequences within each message sequence. Probe sets were located in the following regions of each mRNA species: RANKL (NM_057149), OPG (NM_012870), M-CSF (NM_023981). Housekeeping genes were: Glyceraldehyde-3-phosphate dehydrogenase (GAPDH; NM_017008), Glucuronidase,, beta (NM_017015) and hypoxanthine phosphoribosyltransferase 1 (HPRT1;NM_012583). Specific mRNA transcripts were captured to specific fluorescent beads by hybridization to capture probe-extender probe interactions. The signal from each hybridized unit was amplified by attachment of biotinylated label probes at multiple binding sites on the complexes, which in turn bound to streptavidin-conjugated R-phycoerythrin (SAPE) to produce fluorescence. The fluorescent signals associated with individual capture beads were read using a Luminex 100 IS system (Luminex Corp., Austin, TX, US) with the bead signature designating RNA target and the SAPE signal designating abundance. For each well, the total fluorescence from each individual bead type (corresponding to individual mRNA species) minus background fluorescence for that bead type was normalized to the geometric mean of the fluorescence of the 3 housekeeping genes also in that well. The normalized signals for individual mRNAs from triplicate wells were averaged to yield a single value for each mRNA species being measured.

### Statistical analysis

For normally distributed data, the statistical test used was ANOVA followed by Tukey’s hsd. Data that was not normally distributed data was analyzed using Mann–Whitney followed by Kruskal-Wallis test if more than two groups were compared. The level of significance was taken as p <0.05

## Results

### Body weight

**Figure 1:**
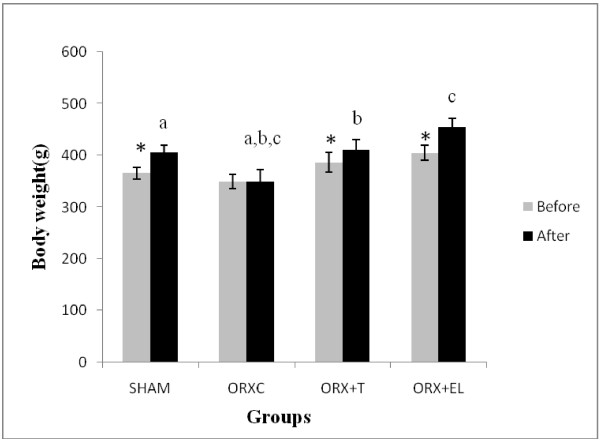
** Mean rat weight before and after treatment.** Data were presented as mean ± SEM with significant difference p < 0.05. *indicate significant difference before and after treatment. Same alphabet indicate significant difference between the groups after treatment. SHAM : Sham- operated group. ORX : Orchidectomized control group. ORX + T : Orchidectomized group treated by testosterone. ORX + EL : Orchidetctomized group treated with EL.

There was a significant weight gain at the end of the treatment period for all the groups except for the ORXC group. Following 6 weeks of treatment, the final bodyweight of the ORXC group was significantly lower than the rest of the groups (Figure 1).

### Testosterone level

**Figure 2:**
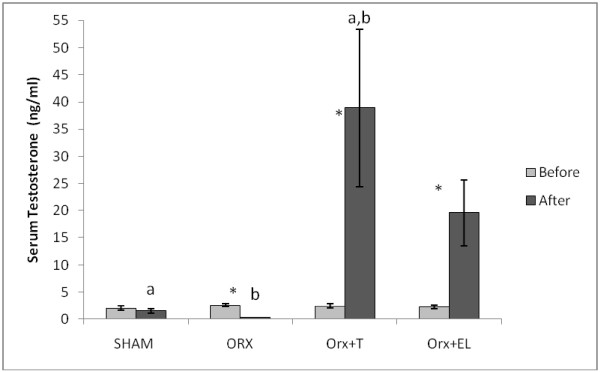
** The testosterone levels before and after treatment.** Data were presented as mean ± SEM with significant difference p < 0.05. *indicate significant difference before and after treatment. Same alphabet indicate significant difference between the groups after treatment. SHAM : Sham- operated group. ORX : Orchidectomized control group. ORX + T : Orchidectomized group treated by testosterone. ORX + EL : Orchidetctomized group treated with EL.

Before treatment, there was no significant difference in the testosterone levels between the groups. After 6 weeks of treatment, the serum testosterone level of the ORXC group was significantly lower than its pre-treatment level. Orchidectomised rats receiving testosterone replacement (ORX + T) or EL supplementation (ORX + EL) had significantly higher post-treatment levels of testosterone compared to their respective pre-treatment levels. When the post-treatment testosterone levels of the different groups were compared, the ORX + T group had significant higher level than the SHAM and ORXC groups but not significantly different compared to the ORX + EL (Figure 2).

### Bone biochemical markers

**Figure 3:**
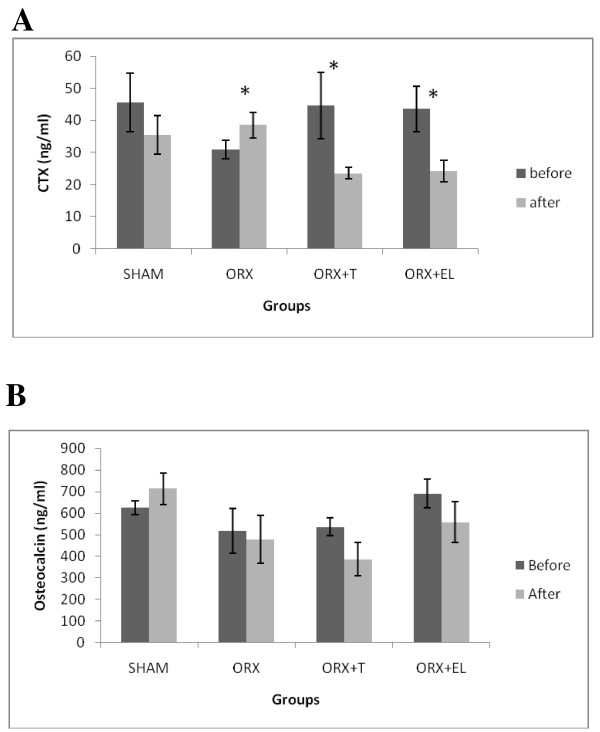
** A and B The serum C-terminal telopeptide of type 1 collagen (CTX) and osteocalcin levels before and after treatment for all the groups.** Data were presented as mean ± SEM with significant difference p < 0.05. *indicate significant difference before and after treatment. SHAM : Sham- operated group. ORX : Orchidectomized control group. ORX + T : Orchidectomized group treated by testosterone. ORX + EL : Orchidetctomized group treated with EL.

The post-treatment level of CTx for the ORXC group was significantly elevated compared to its pre-treatment level. In contrast, there was significant reduction in the post-treatment levels of CTx for the ORX + EL and ORX + T groups compared to their respective pre-treatment levels (Figure 3A). There were no significant changes in the osteocalcin levels for all the groups (Figure 3B).

### Biomechanical test

**Figure 4:**
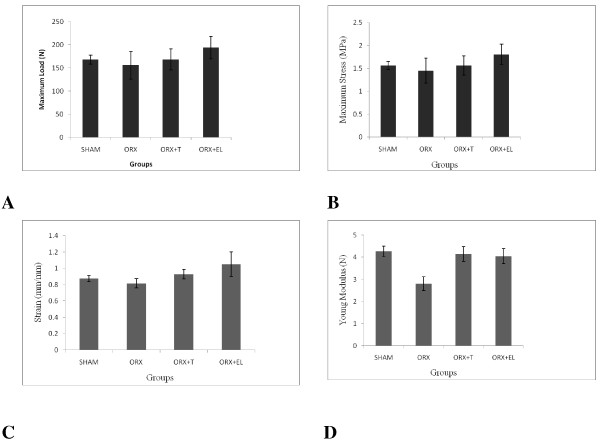
**A, B, C and D: The Load, Stress, Strain and Young Modulus for all the groups.** Data were presented as mean ± SEM with significant difference p < 0.05. SHAM : Sham- operated group. ORX : Orchidectomized control group. ORX + T : Orchidectomized group treated by testosterone. ORX + EL : Orchidetctomized group treated with EL.

There were no significant differences in all the biomechanical parameters for all the groups, even though the Young modulus appeared to be reduced by orchiectomy and revived by testosterone or EL supplementation (Figures 4A–D).

### Gene expression

**Figure 5:**
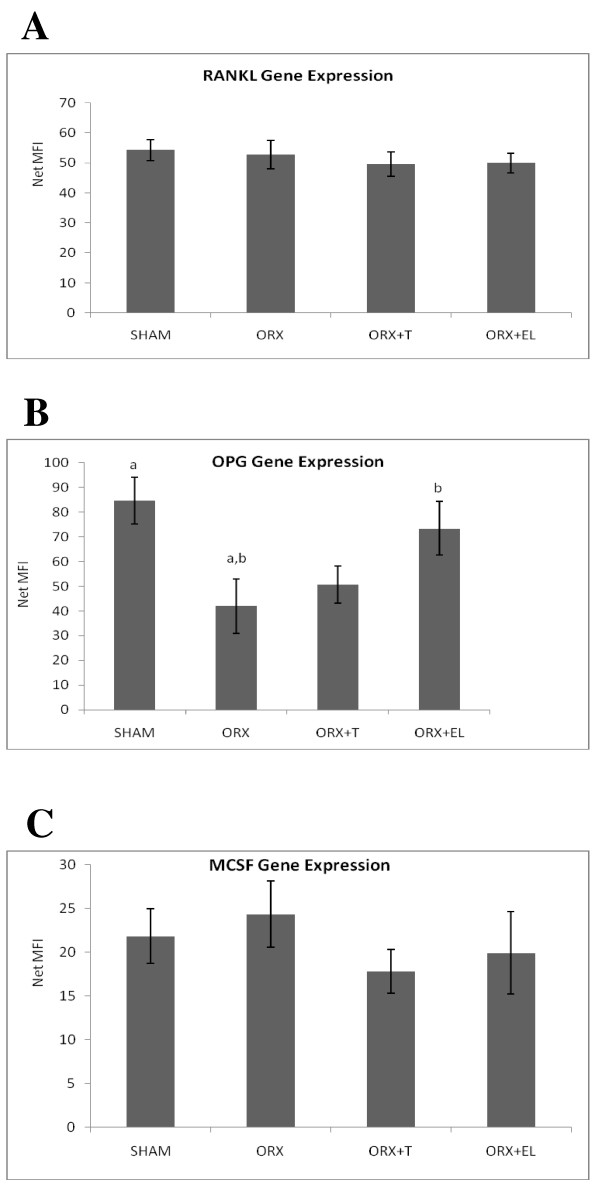
** A, B and C: The RANKL, OPG, M-CSF gene expressions for all the groups.** Samples were normalized to GAPD MFI (Median Fluorescence Intensity). Error bars represented the standard deviations of the average responses. Significant difference was taken at p < 0.05. SHAM : Sham- operated group. ORX : Orchidectomized control group. ORX + T : Orchidectomized group treated by testosterone. ORX + EL : Orchidetctomized group treated with EL.

The RANKL gene expression of the tibial bones for all the groups was not significantly different from each other (Figure 5A). The OPG gene expression of the tibial bone was decreased after orchiectomy as shown by the significant drop in the OPG gene expression of ORX compared to SHAM groups. EL supplementation (ORX + EL) was able to increase the OPG gene expression back to SHAM level. Testosterone therapy (ORX + T) did not cause any significant difference in the OPG gene expression compared to the other groups (Figure 5B). The MCSF gene expression of the rat femur seemed to be increased after orchiectomy (ORX) and reduced with EL supplementation (ORX + EL) and testosterone therapy (ORX + T). However, the difference were not significant (Figure 5C).

## Discussion

The orchidectomised rats in our study failed to gain weight normally until its body weight was significantly lower than the rest of the groups. Testosterone reduction due to orchiectomy may have caused the weight loss due to loss of muscle and bone mass [31]. Testosterone and EL extract were able to prevent these reductions and therefore maintained the weight gain of the orchidectomized rats.

Orchidectomized rat is an accepted model for studying the skeletal effects of androgen deficiency in hypogonadal men [26]. As expected, the testosterone levels of the orchidectomised rats in our study were significantly reduced while testosterone therapy significantly raised the testosterone levels. Similar results were obtained by other studies whereby orchiectomy had caused 80% reduction in serum testosterone in male rats [32], while testosterone replacement was able to raise the testosterone levels [33]. Interestingly, EL extract supplementation to orchidectomized rats at 15 mg/kg for 6 weeks was able to elevate the serum testosterone level significantly. The only published report on the effects of EL on testosterone levels was by Zanoli et al. [34]. They found that supplementation of EL extract to adult male *Sprague–Dawley* rats at 500 mg/kg for 6 days had significantly raised the testosterone levels compared to unsupplemented rats. The dose given to the intact rats by Zanoli et al. [34] was very much higher than ours, but the duration of treatment was shorter. A daily dose level of 270–350 mg/kg was found to be rather safe without any effects on the liver, kidneys, spleen, and testes [35].

The question now is how EL extract was able to raise the testosterone levels in the absence of the testes in our study. In rats, there are other extra testicular sites that synthesize androstenedione and testosterone such as the liver, kidneys and gastro-duodenal tract [36] but not the adrenal glands [37]. Besides that, there was significant formation of 20α-dihydropregnenolone, the precursor of testosterone, in all rat tissues. The enzymes involved in testosterone synthesis such as 3β-hydroxysteroid dehydrogenase, 17β and 20α-hydroxysteroid dehydrogenases were also expressed extra-glandularly in rats [36]. In humans, serum testosterone was detected above the sensitivity threshold even in patients who had undergone surgical bilateral orchiectomy. There is possibility that testosterone production is upregulated in those organs other than testis in surgically castrated patients with detectable serum testosterone measurement [38].

All these findings indicated that there will still be testosterone production even in the absence of testes. There is a possibility that EL extract was able to upregulate these extra testicular testosterone production in orchidectomised rats. More importantly, the testosterone-raising ability of EL extract may be responsible for its bone protective effects in orchidectomised rats.

Testosterone levels may vary between subjects and individuals. This was seen in mice of the same age and strain housed under identical conditions with the testosterone levels ranging from 1 ng/ml to 30 ng/ml. A two to five fold differences in plasma testosterone levels were recorded between samples collected from the same animal at different times [39]. The wide variation in testosterone levels was also seen in human males, ranging between 250 to 850 ng/dl [31]. The testosterone levels fluctuate throughout the day as it peaks in the morning and then gradually dropping throughout the day. We have attempted to reduce these variations by taking the blood samples at the same time at 9.00 am in the morning, when the testosterone levels were high. The testosterone levels also deteriorated with aging whereby its level dropped to half in a 60-year-old man compared to a young man [40].

Major changes in bone mass and micro-architecture can be observed four weeks post-orchiectomy and these alterations become more obvious after four months [41]. The duration of treatment in our study was six weeks as in previous study by Shuid et al. [8]. It should be sufficient for orchiectomy to produce significant bone changes. This was reflected by an elevated CTx level after orchiectomy, indicating increased rate of bone resorption. The elevation of CTx level was suppressed by EL extract supplementation and testosterone therapy. Therefore, both *EL* supplementations and testosterone therapy were able to prevent the increased bone resorption rate seen after orchiectomy. The results not only confirmed the findings by others that testosterone reduced bone resorption markers [9-11], but also confirmed an earlier study by Shuid et al. [8] that EL extract was capable of doing the same.

The bone resorptive changes were not accompanied by any significant changes in the biomechanical parameters. Previous studies have also failed to detect any significant change in the bone biomechanical properties of orchidectomised rats [42,43]. The bone strength is determined by the bone mass and the intrinsic properties of the bone material [44,45]. It is likely that there was no significant mechanical change in the organization of the bone matrix of the orchidectomised rat that may compromise the bone strength.

Since the bone marker changes in this study and another previous study by Shuid et al., [8] were indicative that orchiectomy only affected the bone resorptive activity of osteoclast, we have studied the gene expressions of OPG, RANKL and MCSF. These factors play an important role in the osteoclast recruitment and activation. We found significant results only for the gene expression of OPG. Orchidectomy had down-regulated the OPG gene expression, which was elevated back to sham level with EL extract supplementation. However, testosterone therapy failed to emulate EL’s action. This suggested a novel regulation of OPG by EL, which may help us to understand the mechanism in protection against androgen-deficient bone loss. There were *in vitro* studies with mixed results on the effects of testosterone on OPG expression. Chen et al. [46] found that testosterone was able to amplify the OPG mRNA expression in a mouse bone-cell culture and in an osteoblastic cell line. In contrast, Hofbauer and Heufelder [47] demonstrated that androgens inhibited OPG production by mature osteoblast, marrow stromal cells and murine marrow stroma.

When the RANKL gene expression was measured in this study, there was no significant difference in the gene expression for all the groups. Proell et al., [48] found that the RANKL expression was increased in the bone marrow of orchidectomised rats, while testosterone was able to return the RANKL gene expression to sham control levels. However two other *in vitro* studies did not find any association between RANKL and orchiectomy. Hofbauer and Heufelder [47] reported that the RANKL mRNA was not consistently detected in mature osteoblasts and marrow stromal cells and it was not regulated by androgens. Chen et al. [46] found that testosterone did not affect RANKL mRNA expression in MC3T3-E1 or mouse bone cells.

We did not find any significant change in the M-CSF gene expression after orchiectomy. There were also no significant differences in the M-CSF gene expression between all the groups. *In vitro* study on human endometrial stromal cells found that M-CSF production was dose-dependently enhanced by the addition of testosterone [49].

The present study has pointed out the potential of EL for treatment of male osteoporosis. Theoretically, the prevalence of male osteoporosis should be lower in South East Asian countries where EL is traditionally used as remedy for male wellbeing. There is no credible statistic on the incidence of male osteoporosis in Asian countries that used EL like Malaysia and Indonesia. By comparing the hip fracture incidence in men, the incidence is 88 and 114 per 100 000 in Malaysia and Thailand respectively [50]. The incidence is 362 and 311 per 100 000 in Norway and Denmark respectively [51]. Therefore the incidence is much lower in countries that used EL. However, the correlation with EL use is difficult to determine as the percentage of men taking EL is not known. Assuming that a significant number of men were taking EL in South East Asia compared to Scandinavia, it would explain why the prevalence of hip fracture in men in South East Asia was only one fourth to one third compared to Scandinavia.

## Conclusions

It can be concluded that both EL extract and testosterone are effective in elevating the testosterone levels, which may explain the bone-protective effects of EL. However, since only EL extract has affected OPG expression, this may be an additional mechanism of EL in protecting against bone resorption induced by androgen-deficient. Further studies on the regulation of OPG production by EL may provide insight into this novel mechanism.

## Competing interests

The author(s) declare that they have no competing interests.

## Authors’ contributions

ANS and INS designed the study. EA, NME and HSAR carried out the study and collected the samples. NM and NM participated in the statistical analysis. All authors read and approved the final manuscript.

## Pre-publication history

The pre-publication history for this paper can be accessed here:

http://www.biomedcentral.com/1472-6882/12/152/prepub
